# The complete mitochondrial genome of the Oriental sea slug: *Chromodoris orientalis* (Nudibranchia, Chromodorididae)

**DOI:** 10.1080/23802359.2018.1508381

**Published:** 2018-10-26

**Authors:** Cheol Yu, Hana Kim, Hyung June Kim, Yun-Hwan Jung

**Affiliations:** a Department of Taxonomy and Systematics, National Marine Biodiversity Institute of Korea, Chungcheongnam-do, Republic of Korea;; b Department of Oceanography and Ocean Environmental Sciences, Chungnam National University, Daejeon, Republic of Korea;; c Department of Biological Sciences, Inha University, Incheon, Republic of Korea

**Keywords:** Mitochondrial genome, *Chromodoris orientalis*, Chromodorididae, oriental sea slug, Korea

## Abstract

The mitogenome sequence of sea slug, *Chromodoris orientalis* (Nudibranchia, Chromodorididae), has been decoded for the first time by coverage genome sequencing method. The overall base composition of *C. orientalis* mitogenome is 30.5% for A, 14.7% for C, 18.0% for G and 36.9% for T, and has low GC content 32.6%. The assembled mitogenome, consisting of 14,266 bp, has unique 13 protein-coding genes, 22 transfer RNAs, and two ribosomal RNAs genes. The *C. orientalis* mitogenome has the common mitogenome gene order and feature of Nudipleura (a clade of sea slugs and sea snails). The complete mitogenome of *C. orientalis* provides essential and important DNA molecular data for further phylogenetic and evolutionary analysis for sea slugs and sea snails.

The genus *Chromodoris* Alder & Hancock, 1855 contains 62 species that is widely distributed throughout the global oceans, including the temperate, tropical, and the Antarctica waters (Rudman 1983; Xiang et al. 2016; Lin et al. 2017; MolluscaBase 2018). Far Eastern waters including Korea and Japan are considered to be the origin of *C. orientalis* so that this species is called an oriental sea slug and they are currently expanding its range of geographical distribution to the Hong Kong sea areas (Hong et al. 2006). In this study, we determined the complete mitochondrial genome of *C. orientalis,* fourth in the genus *Chromodoris*.

Specimens of *C. orientalis,* collected from a SCUBA diving of rocky subtidal, in Uljin-gun, Gyeongsangbuk-do, East Sea of Korea. The voucher specimen was deposited in National Marine Biodiversity Institute of Korea (MABIK MO00171931). The genomic DNA was extracted from the muscle tissue by DNeasy Blood & Tissue Kit (QIAGEN) and its protocol. The mitogenome sequences were analyzed by application of Illumina Hiseq2000 sequencing platform (Macrogen, Seoul, Korea). These sequences were annotated in comparison with mitogenome sequences of species belonging to the order Nudibranchia previously reported by Lin et al. (2017) using Geneious 9.1.8 (Kearse et al. 2012). Additionally, we used the mitochondrial genome annotation (MITOS) server (Bernt et al. 2013) and tRNAscan-SE server (Lowe and Chan 2016) for annotation. Neighbor-Joining (NJ) analysis was constructed using K2P model in MEGA6 (Tamura et al. 2013) and dataset were used nucleotide sequences of 13 protein coding genes (PCGs).

The circular mitogenome of *C. orientalis* is 14,266 bp in length (GenBank accession number MH550543) with 13 PCGs, two ribosomal RNAs (rRNAs), and 22 transfer RNAs (tRNAs). The mitogenome of *C. orientalis* has a high A + T content (67.4%) similar to the other *Chromodoris* species (A: 30.5%; C: 14.7%; G: 18.0%; T: 36.9%). 11 PCGs (*cox1, nad6, nad5, cob, cox2, atp8, atp6, nad3, nad4, cox3,* and *nad2*) get off by the typical ATG start codon, but two PCGs (*nad1* and *nad4l*) use ATT and ATA as start codon, respectively. Eight PCGs (*cox1, nad6, nad4l, cox2, atp8, atp6, nad3,* and *nad4*) use TAA for the stop codon and five PCGs (*nad5, nad1, cob, cox3,* and *nad2*) ends with TAG. The lengths of tRNA genes range from 56 to 70 bp and all tRNAs have the typical clover leaf structure except two tRNA^Ser^_UCN_ and tRNA^Ser^_AGC_ reduced DHU arm.

As a result of confirming the molecular phylogenetic position about this species was closely related to other *Chromodoris* species and was grouped within family Chromodorididae (Figure 1). The mitogenome of *C. orientalis* will be useful for inferring the phylogenetic relationships among the members of Chromodorididae within the Nudibranchia.

**Figure 1.**
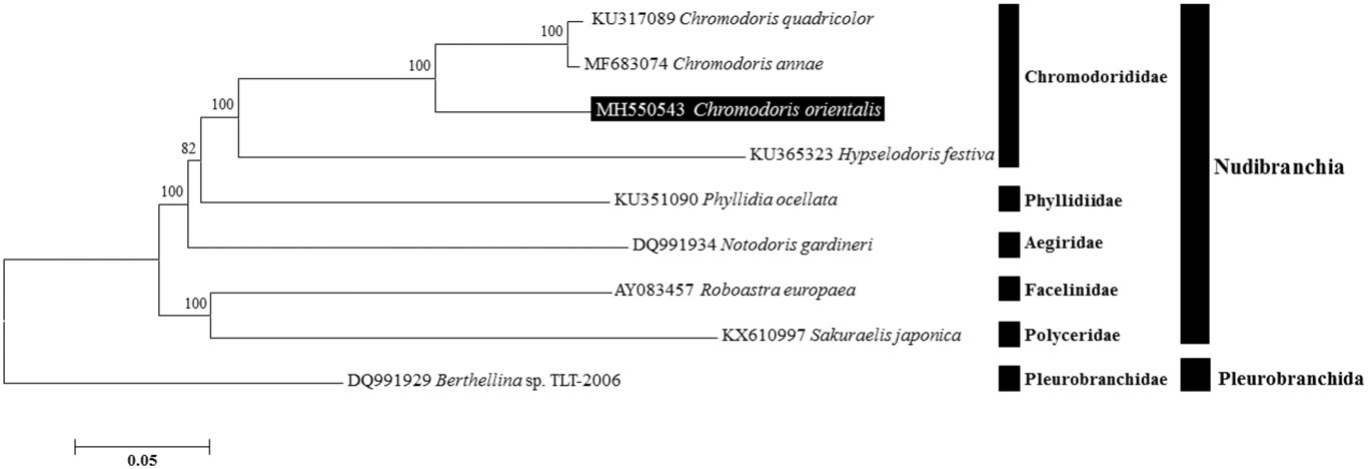
Neighbor-Joining (NJ) tree based on the protein coding genes (PCGs) of *Chromodoris orientalis* and other sea slugs under order Nudibranchia. *Berthellina* sp. derived from Pleurobranchida was used as outgroup for tree rooting. Numbers above the branches indicate NJ bootstrap values from 1,000 replications.
